# Differences in boldness between Eurasian and American wolves (*Canis lupus*) might be based on adaptive mechanisms

**DOI:** 10.1002/ece3.70178

**Published:** 2024-08-19

**Authors:** Hana Tebelmann, Udo Ganslosser

**Affiliations:** ^1^ Institute of Biology and Environmental Sciences, School of Mathematics and Science Carl von Ossietzsky Universität Oldenburg Oldenburg Germany; ^2^ Institute for Zoology and Evolutionary Research, Faculty for Biosciences Friedrich Schiller Universität Jena Jena Germany

**Keywords:** animal behaviour, animal personality, boldness, cooperation, environmental adaption, novel object interaction

## Abstract

Boldness – defined as the propensity of individuals to take risks – is a key research area within animal behavioural studies, significantly affecting adaptive strategies, habitat selection, foraging behaviour, reproduction, dispersal, and other crucial survival behaviours. Despite the extensive study of personality traits like extraversion and curiosity across various animal species, data on wolves (*Canis lupus*), particularly on the subspecies level, remains sparse. This study aims to bridge this gap by examining boldness and its associated personality traits in different wolf subspecies (*Canis lupus lupus*, *Canis lupus arctos, Canis lupus lycaon*) (*n* = 23), and wolf‐dog hybrids (*n* = 10), utilising novel object interaction tests and validated questionnaires previously applied to wild canids. Our results show significant differences in boldness as well as in related personality traits between taxa, both between pure wolves and wolf hybrids, with significantly higher boldness of North American subspecies. The inter‐subspecies differences were more significant than the differences between groups or at the individual level, suggesting that subspecies ecology and historical selection pressure in subspecies history might have caused long‐lasting adaptations in *Canis lupu*s ssp.

## Graphical abstract

Boldness – defined as the propensity of individuals to take risks – is a key research area in animal behaviour; however, some species, like wolves, are sparsely studied. We investigated differences in bold behaviour and its associated personality traits in different subspecies of captive wolves. We found significant differences in boldness as well as in related personality traits between taxa, both between pure wolves and wolf hybrids, with more significant inter‐subspecies differences than within‐group or individual differences.

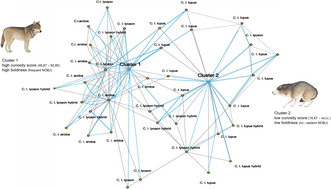

## 1 INTRODUCTION

Animals often differ in their propensity to take risks (“boldness”) in a range of functional contexts related to fitness, e.g. dispersal and foraging (Beckmann & Biro, 2013; Chapman et al., 2010, 2011; Dammhahn & Almeling, 2012; Wilson et al., 1993). When presented with a startling stimulus or a novel object, individuals may differ consistently in their responses over repeated observations (Stamps & Groothuis, 2010a, 2010b). Consistent individual differences in behaviour (“animal personality”) might appear as a result of certain ecological conditions, most notably predation and social environment (Sih et al., 2015). Personality and behavioural syndromes can evolve in response to selection (Bell, 2007), implying that syndrome structure could differ between selective environments or populations depending on their evolutionary history of selection. The degree of bold behaviour in a population can be affected by environmental influences (Carere & van Oers, 2004). According to Sinn et al. (2008), it is likely that instructive developmental processes leading to bold or shy behaviour have evolved themselves. Individuals within a population consistently respond to stressors in a particular manner, and some responses are more beneficial than others, the environment may influence which behavioural tendencies dominate in a population, habitat or even a species within a certain distribution range (Robertson, 2018). Exogenous traits have recently come to the fore as key state variables influencing variation between individuals (see Dingemanse & Araya‐Ajoy, 2015). Environmental conditions, such as historical anthropomorphic influences, can exert diverse selection pressures on different behaviours (Dall et al., 2004, 2012; Garamszegi et al., 2012; Sih & Bell, 2008; Sih et al., 2015; Wolf & Weissing, 2012). Behavioural types might be preserved in certain populations, either because natural selection induces correlational selection (see Dingemanse & Réale, 2005) or because strong pleiotropic effects of hormones or genes have resulted in syndromes that cannot easily be modified during evolution (‘constraint hypothesis’; Bell, 2007). For the shaping of personality traits through evolutionary and ontogenetic processes, four mechanisms have been proposed: nongenetic inheritance of personality (Guerrero‐Bosagna et al., 2018; Reddon et al., 2012), genetic inheritance of personality (Ariyomo et al., 2013; Biro & Stamps, 2008; Dochtermann et al., 2015; van Oers et al., 2005), gene–environment interactions (reaction norms) shaping the development of personality (Dingemanse et al., 2003; Dochtermann et al., 2015; Réale et al., 2007; Schuett et al., 2013; Wiese et al., 2018; Wolf & Weissing, 2012), and positive feedbacks between individual state and behaviour (Sih et al., 2015). In this paper, we will focus on nongenetic behavioural inheritance as well as gene–environment interactions.

Personality traits have been found in a wide range of species including, inter alia, arthropods (Johnson & Sih, 2005), birds (Dingemanse et al., 2004), amphibians (Carlson & Langkilde, 2013), fish (Burns, 2008), reptiles (Clobert et al., 2000), and mammals (Réale et al., 2009). Behavioural consistency is a fundamental part of the definition of personality (Cabrera et al., 2021).

Where research on wild animals is difficult, investigating the behaviour of captive animals can help to understand mechanisms that are relevant in wildlife and in general species ecology. It remains a challenge to grasp how variation in traits that are under selection is maintained in the wild (Freeman & Herron, 2007).

Inter‐individual differences (Soriano et al., 2021) and behavioural consistency (Edwards, 2018; MacDonald, 1983) have only been sparsely studied in wolves, particularly across populations or subspecies. In Europe, wolves commonly show a tendency to avoid anthropogenic habitats (Karlsson et al., 2007; Ordiz et al., 2015), and they tend to select areas further from anthropogenic features (Milleret et al., 2019). In Eurasia, thresholds for settlements and roads where wolves are not present are significantly higher compared to North America (Sazatornil et al., 2016). In North America and Arctic regions, wolves live in mostly remote areas in large national parks or pristine environments, where they colonise forested areas relatively far from human civilisation (Jędrzejewski et al., 2008; Mladenoff et al., 1995; Oakleaf et al., 2006). Wolves' reactions to human activity are shaped by the cumulative sum of repeated interactions with humans and the consequences of these interactions (Bejder et al., 2009; McNay, 2002; Whittaker & Knight, 1998). Human impacts (e.g. persecution, disturbance) can lead to different behavioural responses by large carnivores, such as wolves, to minimise their interactions with humans and thereby the impacts of such (e.g., Ahmadi et al., 2014; Lesmerises et al., 2013; Llaneza et al., 2012; Whittington et al., 2005). We therefore assume that wolves (i.e., North American wolves) with a species history of fewer negative experiences with anthropogenic stimuli, such as unfamiliar objects, exhibit significantly less neophobia and increased boldness.

*Canis lupus* is often considered only as a species, but not population‐ or subspecies‐specific, despite major differences in eradication history between the Eurasian and American continents. In this paper, we elucidate the inter‐subspecies behavioural variation in captive wolf subspecies with differing environmental influences and exploitation histories. We studied several wolf groups of different subspecies, split into North American wolves and Eurasian wolves, in relation to their reaction to novelty and personality traits. Neophilia vs. neophobia was measured using novel object interaction (NOBJ) with an apparatus. We hypothesise that the different habitat conditions between Eurasian and North American wolf subspecies, and their hybrids may have had a lasting effect on their behaviour, especially their tendency to explore novelties and display bold behaviour. Our primary hypothesis is that neophilia vs. neophobia, as an indicator of boldness, is significantly influenced by subspecies and their inherent evolutionary and environmental determinants. We examine the extent to which the bold behaviour differs between Eurasian and North American wolves. Additionally, we explore the relationships between species, age, sex, rearing conditions, and personality traits in wolves to answer the question: “Who's the ‘big, bold wolf’?”

## 2 METHODS

### 2.1 Ethical note

No licenses or permits were required for this research. During trials the wolves were not harmed. All the animals remained in their permanent enclosure and no direct contact between humans and animals took part.

### 2.2 Study species and animal care

**FIGURE 1:**
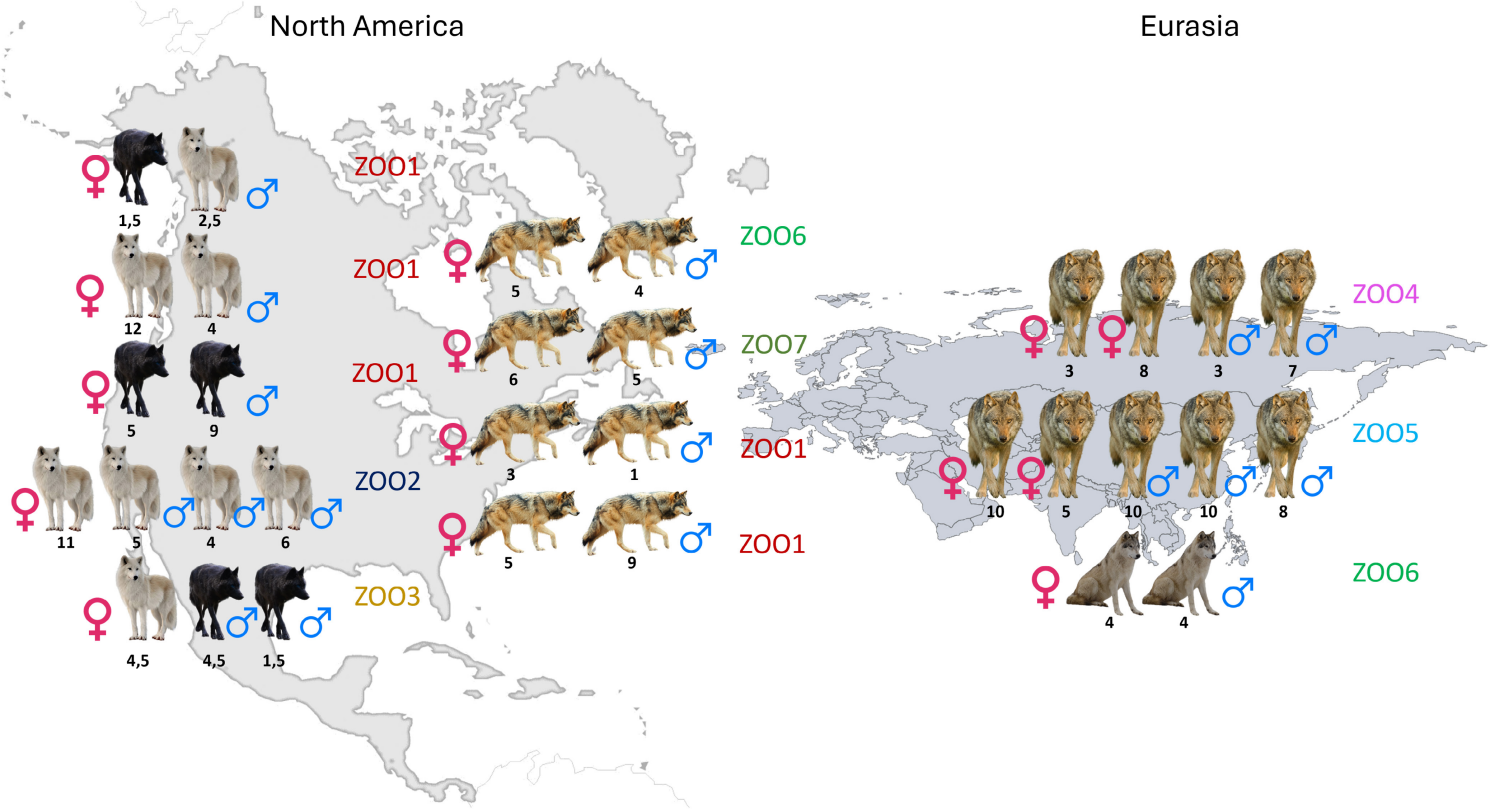
Graphical representation of the included groups. The animals are organised according to geographical subspecies (North American wolves and North American wolf‐dog hybrids on the left, Eurasian wolves and Eurasian wolf‐dog hybrids); animals that are shown next to each other each represent a group. The colour of the animals indicate their subspecies, black wolves are C*anis lupus lycaon*, white wolves are Canis lupus arctos, grey wolves in North America are hybrids. Grey wolves in Eurasia are *Canis lupus lupus*. The sex icon (female on the left, male on the right) indicates the respective sex of the animals. “ZOO” with respective colour indicates the facility in which the animals were kept at the time of the trial. The black numbers below the animals represent the age (in years) of the respective animal.

Seven groups of *Canis lupus* (*Canis lupus lupus* (*n* = 9), *Canis lupus arctos* (*n* = 8), and *Canis lupus lycaon* (*n* = 5)) as well as five groups of wolf‐dog hybrids (F2‐F3, *Canis lupus lycaon* × *Canis familiaris* (n = 8) and F3, *Canis lupus lupus* × *Canis familiaris* (*n* = 2)) with 2–5 individuals per group were observed for the study (see Figure 1). We have included both pure wolves of different subspecies from the North American region as well as from Eurasia and wolf‐dog hybrids, given that wild traits are strongly preserved and conserved in low filial generation hybrids (Fox, 1975; Hansen Wheat et al., 2018; Lescureux, 2018; Zimen, 1987). We, therefore, tested if behavioural profiles of wolf‐dog hybrids of distinct wolf subspecies would differ from pure wolves. The animals within groups, but not pairs, were related, consisting of natural packs (breeding pair and offspring). There was no relatedness between animals of different groups. All animals have been bred and raised in captivity. Out of all animals, 5 were hand reared. The age of the animals ranged from 1 to 11 years (6.57 ± 2.91, see Table A1). All groups included male and female wolves or wolf‐dog‐hybrids (male = 18, females = 14). None of the animals had prior experience in similar behavioural research. Within our North American subspecies groups, *Canis lupus lycaon* and *Canis lupus arctos* were kept together in two of five groups. All animals remained in their permanent enclosures in the respective zoological facility or sanctuary. We observed animals at different facilities in Germany (Game Park Schwarze Berge, Wolf Sanctuary, Zoo Wingst, Bear Park Worbis, and Game Park Petersberg), Sweden (Vasternorrlands Lan) and the U.S. (Wild Spirit Wolf Sanctuary).

### 2.3 Experimental set‐up and procedure

**FIGURE 2:**
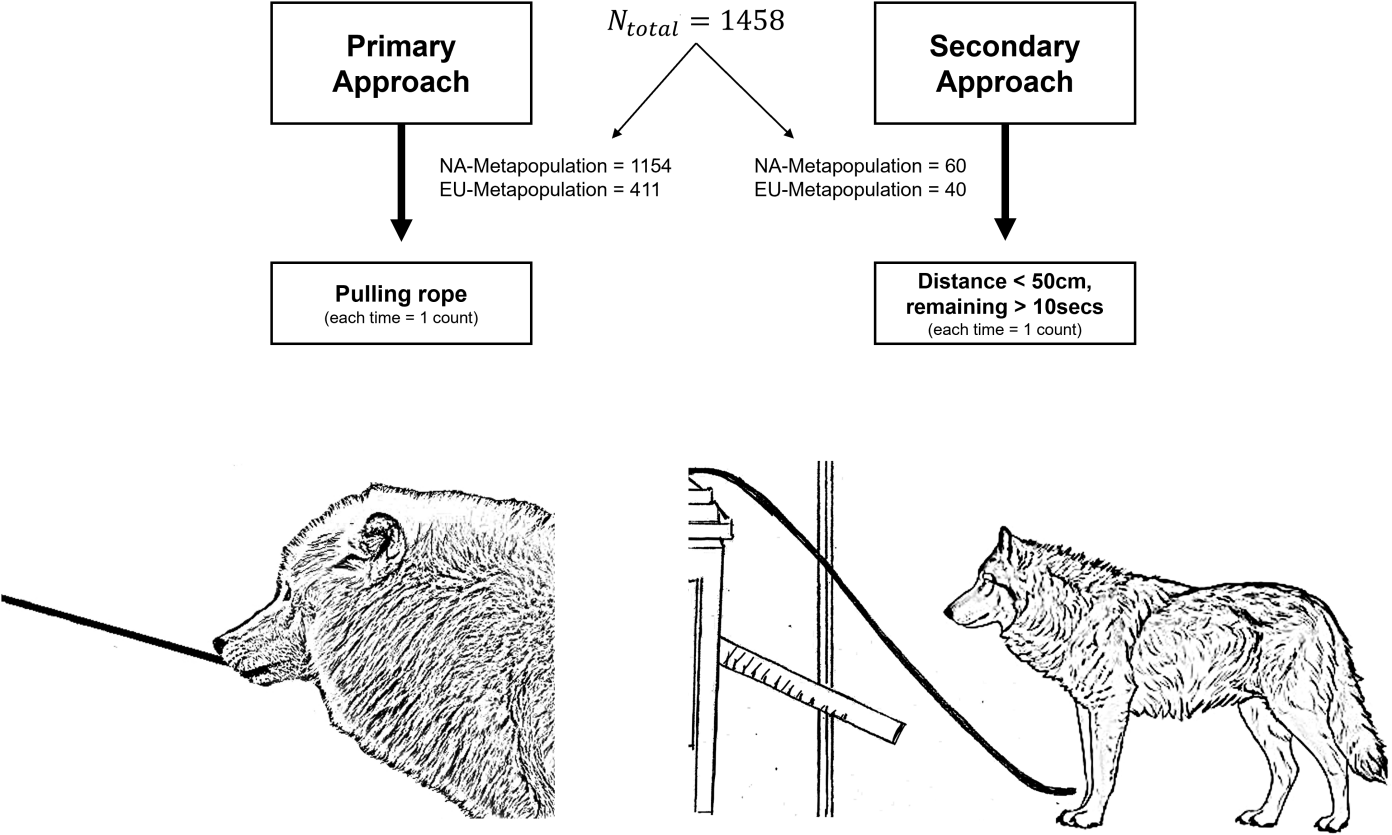
Illustration of the experimental set‐up displaying primary and secondary approaches. Ntotal represents the total number of observations of NOBJ. NA taxa include *Canis lupus lycaon*, *Canis lupu*s *arctos* and the hybrids with dogs, EU taxa *Canis lupus lupus* and hybrids with dogs. Primary approaches were direct physical contact, i.e. pulling the rope or the tubes, A secondary approaches were close proximity to the apparatus (below 50 cm for a minimum of 10 s). Each primary approach was recorded as one count. Each iterative secondary approach was recorded as one count.

We observed the tendency of novel object interaction (NOBJ) using an apparatus. Each individual approach to the apparatus was rated as a “novel object interaction” (see Figure 2). Approaches were coded into primary and secondary approaches. As a primary approach, direct physical interaction with the apparatus, i.e. pulling the rope or the tubes, was recorded. A secondary approach was defined as being in close proximity (below 50 cm for a minimum of 10 s). Each primary approach was recorded as one count. Each iterative secondary approach was recorded as one count.

To accustom the animals, the apparatus was initially placed in front of the enclosure for at least two weeks, and the observer was positioned in front of the enclosure for several hours during the pre‐observation phase.

Data could not be recorded blindly because our study involved focal animals in the field.

During the trial, the apparatus was placed in or in front of the enclosure. The tubes and ropes were led through the fence at the time of the trial and were removed thereafter to avoid unsupervised use. Outside the trial period, the apparatus was stored within sight of the animals a few metres away from the enclosure. The trial was conducted over a period of 21 days per animal group plus a pre‐observation phase. Two trials were made per day per group with a total number of 252 observation days (1.512 h, *n* = 1458). The overall experimental period took 2–4 months per group with observations up to 4 times per week per group. All groups had regular breaks between trials for at least one day between trials. All trials were conducted using the same type of apparatus, specifically build for each group to prevent avoidance due to scent of unfamiliar animals.

Before the start of the trial, the apparatus was installed in front of the enclosure of each group for a habituation period of two weeks. The pre‐observation phase served both to accustom the animals to the observer and to ensure that the observer can distinguish the individuals. Throughout the trial, the apparatus was installed in front of each group's enclosure with the tubes sticking through the enclosure fence.

### 2.4 Behavioural observations on novel object interaction

During our observations, the animals had the opportunity to approach or use the apparatus (secondary vs. primary approach). The use of the apparatus (primary approach) had fitness consequences, as the animals were able to obtain food for themselves as well as for their conspecifics. The subspecies of Eurasian and North American wolves differed considerably in the number of approaches, especially their primary approaches. The North American taxa (*Canis lupus lycaon*, *Canis lupus arctos* and hybrids with domestic dogs) showed 1154 primary and 60 secondary approaches, the Eurasian taxa (*Canis lupus lupus* and hybrids) showed 411 primary and 60 secondary approaches (see Figure 2). Apart from three Eurasian wolves in two different zoos, all tested animals showed both approaches (primary and secondary). Seven individuals, including two from the North American wolf subspecies and five from the Eurasian wolf subspecies, showed no approaches at all. Interestingly, after performing a primary approach, the Eurasian wolves did not show any approach for a longer period of time, neither primary nor secondary. The Eurasian wolves reacted to the use of the apparatus with explicit bodiespeaking signs of fear, both the approaching and observing animals.

### 2.5 Personality assessment

To test for individual, within group and inter‐subspecies differences in behaviour and personality, we combined proven methods. When studying the personality traits and behaviours of a given species, it is of paramount importance to test with the most appropriate variables to record the behaviours (Finkemeier et al., 2018). To assess behaviours in captive animals, there are two main methods: coding the behaviour performed by the animals in experimental (i.e., behavioural test) or non‐experimental situations (ratings of pre‐established traits or ethological coding via ethograms). It is largely accepted that a combination of both methods is the most accurate measure for behavioural traits or personality (Masilkova et al., 2018). Therefore, we used a combined approach of behavioural codings in a novel‐object‐approach test and ethological codings via questionnaires on well‐established personality traits in canids.

To evaluate personality factors, we used the DOGS personality questionnaire (Turcsán et al., 2011) as well as the Monash Canine Personality Questionnaire – Revised (MCPQ‐R) (Ley et al., 2009). We adapted the DOGS questionnaire and the MCPQ‐R for canids living in zoological institutions and modified the questions accordingly (see Table S1). The respective questionnaires were handed out to the zookeepers. The DOGS personality questionnaire includes the factors of trainability, sociability, extraversion, and calmness. The MCPQ‐R contains the factors extraversion, self‐assuredness/motivation, training focus, amicability, and neuroticism. Because the personality factors of the questionnaires are similar, we grouped the factors extra‐version/extraversion and sociability/amicability into personality dimensions. Since the trainability factor is not applicable for wild canids, but relates to intelligence, interest in novelty, and playfulness, we decided to split this factor into two factors: Curiosity and playfulness (see Table S1). The trainability factors of the original questionnaires contain items of playfulness and intelligence and are comparable to the Big Five dimension of openness, which is related to curiosity in humans and animals.

### 2.6 Statistical analysis

#### 2.6.1 Homogeneity of subspecies

We addressed variances in each of our groups by subspecies and group performing a Fligner‐Killeen test of homogeneity of variances (R Studio, ‘stats’ package) for North American wolf subspecies. The test is robust for non‐normally distributed data.

### 2.7 Analysis of frequency of novel object interaction and correlating factors

For a nuanced approach to measuring boldness, primary versus secondary approaches were combined to NOBJ, while primary approach was weighted at 2:3 compared to the secondary approach.

All data analysis was carried out using RStudio Version 4.2.1 (R Core Team, 2023). Data on novel object interaction (NOBJ) were analysed using zero‐inflated Poisson regression models with package ‘glmmTMB’ (Ho et al., 2011). We conducted a causal inference analysis on the frequency of novel object interaction (NOBJ) to estimate the effect of subspecies (North American vs. Eurasian subspecies) on NOBJ. Our initial hypothesis was that subspecies (SSP) exert the primary influence on NOBJ. Curiosity (CRS), age, relatedness, sex, group structure, and hand rearing were considered confounding variables that are related to both the exposure (SSP) and the outcome of interest (NOBJ). We used a propensity score to match observations from our exposure groups to ensure comparability with package ‘MatchIt’ (Brooks et al., 2017). The balance between the matched groups was evaluated by calculating the standardised mean difference (SMD) for each confounder. Based on the propensity scores, we computed inverse probability weights (IPW) for each observation and applied these weights in a weighted zero‐inflated Poisson regression analysis. All models included random intercepts for animal (since the same individuals were measured for multiple days).

We tested for the influence of date as a random effect, since wolves are known to habituate. As date did not have a significant effect in our generalised linear mixed effects model (*R* ^2^–.002, *p* > .5), it was not included as a random effect.

Additionally to our models, we performed a k‐fold cross‐validation to assess clusters within the data. We modelled the distance between individuals via k‐means, Silhouette Clustering and NMDS using packages ‘cluster’ (Maechler et al., 2022), ‘ClusterR’ (Mouselimis, 2023), ‘factorextra’ (Kassambara & Mundt, 2020), ‘caret’ (Kuhn, 2008), ‘NbClust’ (Charrad et al., 2014), and ‘vegan’ (Oksanen et al., 2024) to determine whether (1) individuals cluster based on their frequency of novel object interaction and (2) a cluster is composed of animals from the same zoo or rather individuals from a subspecies.

Additionally, we carried out a Kruskal–Wallis test to account possible inter‐ and intra‐group differences in NOBJ with package ‘psych’ (Revelle, 2024), also conducting pairwise comparisons. Effect sizes for the Kruskal–Wallis test were computed with DurgaDiff (package ‘Durga’, Khan & McLean, 2023).

### 2.8 Temporal stability of bold behaviour

To test whether NOBJ as a potential measure of boldness is temporarily stable, we performed a segmented generalised linear model in package ‘segmented’ (Muggeo, 2008) as well as a time‐series analysis and a forecast. The autoregressive integrated moving average (ARIMA) model with package ‘forecast’ (Hyndman & Khandakar, 2008) was used to demonstrate the time series and predicted development of NOBJ in Eurasian wolves, North American wolves as well as wolf‐dog hybrids for each group from the next 21 days after the automatic selection method of ARIMA parameters. Statistically significant ARIMA parameters from each model were reported with approximate standard errors, and model with the lowest AIC was selected as the best fit for the observed time series. Since the parameters of the groups differed in part, the best model fit was chosen for the respective group. Our other forecast model used a simple exponential smoothing (SES) approach using package ‘fpp2’ (Hyndman & Athanasopoulos, 2018), using weighted averages of past observations, with exponentially decaying weights as the observations get older.

### 2.9 Influence of personality traits

It is known that personality traits are correlated to bold behaviour (Sih et al., 2004; Svartberg, 2005). Boldness and personality traits also differ between animals of different ages and sexes. To determine the influence of personality factors on bold behaviour, we used the scores from the DOGS questionnaire and the MCPQ‐R. To evaluate the data, the raw scores for each adjective within each personality factor subscale were summed and divided by the maximum score possible for the subscale. The result was converted to a percentage, thereby creating a percentage score for each of the five personality factors for every individual. To estimate the correlation between the personality traits, age and sex, a correlation matrix was created, and a multiple correlation analysis using distance correlation coefficient was performed with package ‘energy’ (Rizzo & Szekely, 2023). Additionally, a generalised linear mixed model with Gamma distribution was performed for the influence of age and sex on personality traits. To test for inter‐group differences, a Kruskal–Wallis test was performed for each personality factor.

## 3 RESULTS

### 3.1 Differences in novel object interaction (bold behaviour)

For modelling the influence on NOBJ, we performed a Fligner‐Kileen test on homogeneity in the subspecies *Canis lupus arctos* and *Canis lupus lycaon*. Based on the results (*p* > .1 for subspecies, *p* < .0001 for group), we reassigned *Canis lupus lycaon* and *Canis lupus arctos* and merged them into a subspecies of North American wolves.

**FIGURE 3:**
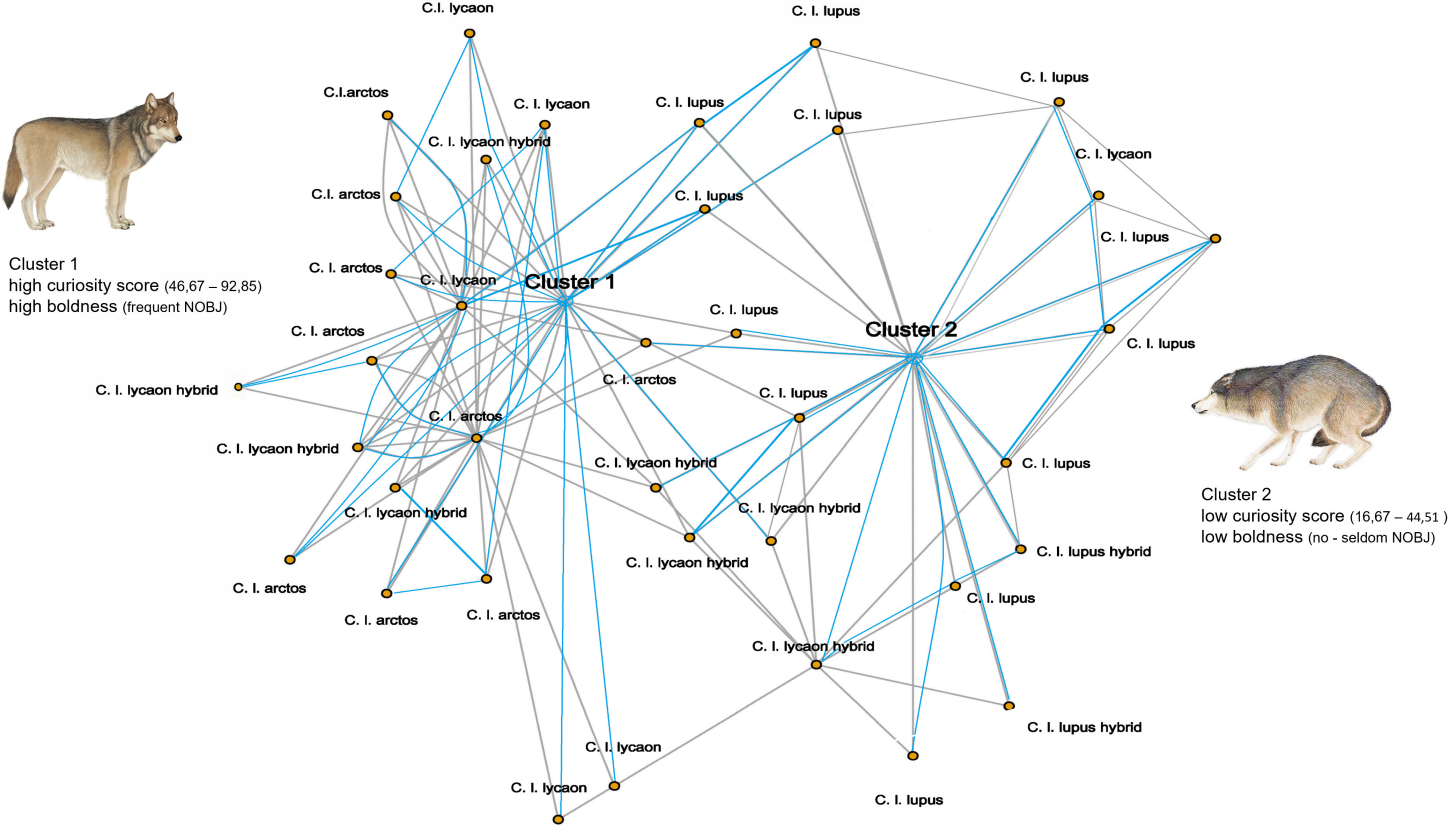
Network plot showing how the individuals clustered in their scores of bold behaviour (indicated by frequency of NOBJ) and curiosity. Blue lines represent curiosity values and connectivity between the individuals within and across clusters, and grey lines represent values for frequency of novel object interaction (NOBJ). Species abbreviations indicate the respective group.

We selected a zero‐inflated Poisson model based on causal inference applied to our matched data (see Table 1). We set Eurasian wolves (SSP) as intercept. Belonging to the North American taxon as well as scoring high in curiosity (CRS) had a highly significant positive effect on the frequency of novel object interaction (*p* < .001), which is natural given the differences in CRS between the taxa (see Figure 3). Zero inflation was influenced by belonging to Eurasian wolves (*p* = .04). The variance in our random effect (animal ID) suggested moderate variability between animals (estimate = 0.3282).

**TABLE 1:** Model output for causal inference models.

Variable	Estimate	SE	*z* Value	*p* Value
Conditional model ouput				
SSPEurasian (Intercept)	−5.03634	1.41206	−3.567	.0001
SSPNorthAmerican	3.93410	0.93830	6.185	.0001
CRS	0.10731	0.01611	6.660	.0001
Age	0.19605	0.07138	2.747	.006
Related	0.87877	1.11044	0.791	.42
Handfed	1.26642	1.06383	1.190	.23
Structurepair	1.15671	0.88116	1.313	.18
Zero‐inflation model output				
SSPEurasian (Intercept)	−0.9808	0.4787	−2.048	.04

*Note*: The conditional model estimated the abundance of NOBJ, where a positive contrast indicates a higher abundance. In contrast, the zero‐inflation model accounted for the probability of an extra zero, where a positive contrast indicates a higher chance of absence.

**FIGURE 4:**
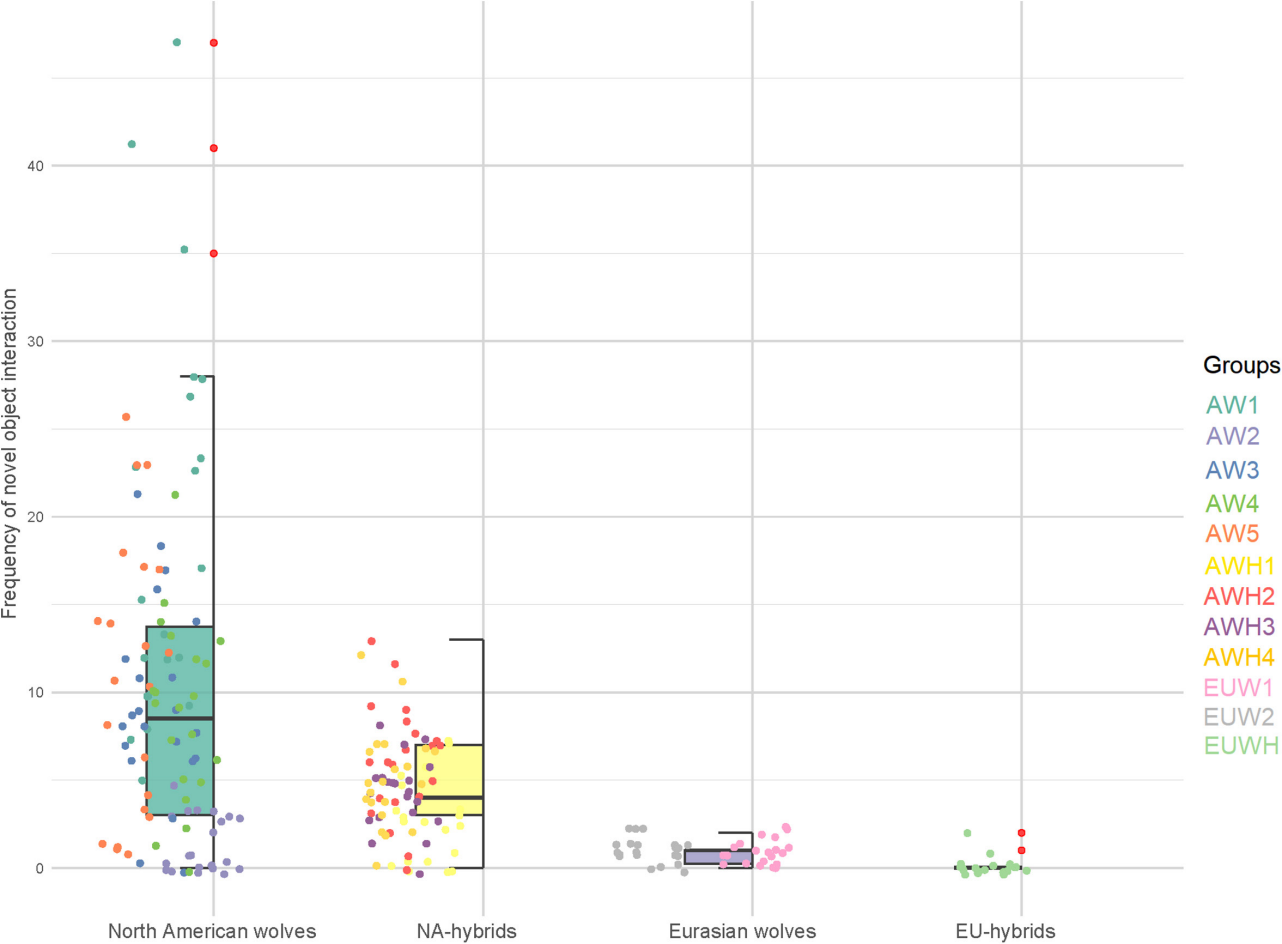
Generalised linear mxed effect model (GLMM) result for the relationship between taxa and frequency of novel object interaction (NOBJ). The differently coloured dots represent the respective data points for each group. Red vertical points indicate outliers. Boxplots represent medians (bar within the box), upper and lower quartiles (borders of box), lowest and highest cases within 1.5 times the IQR (bottom and top whiskers).

Based on our model results, we grouped the respective taxa into two different clusters, consisting of Cluster 1 for individuals with high boldness and high curiosity and Cluster 2 for low boldness and low curiosity. Clusters were created based on the values of NOBJ and curiosity using hierarchical edge bundling, in which individuals are grouped and connected based on the distance of their respective values to other individuals. Close bonds indicate a lower variance in the values for NOBJ and curiosity within the clusters, and vice versa (see Figures 3 and 4).

**FIGURE 5:**
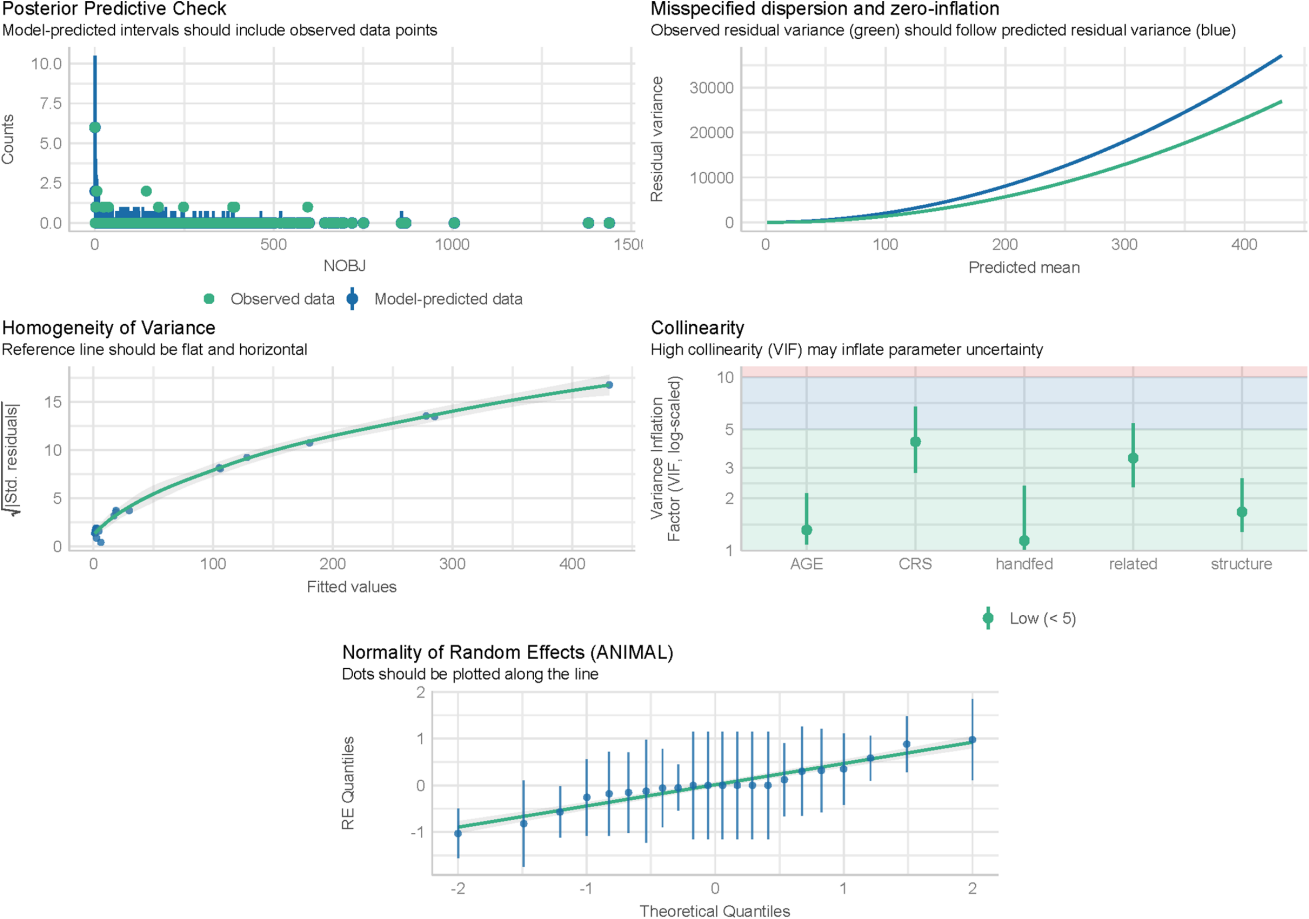
Goodness‐of‐fit model estimation. The posterior predictive check panel illustrates the overlap between observed data points (green circles) and model‐predicted data (blue triangles) across the range of NOBJ values. The residual variance is plotted against the predicted mean to assess the model's ability to capture the underlying data structure; observed residual variance (green) should ideally follow the predicted residual variance (blue). Deviance suggests slight dispersion issues in our model. The homogeneity of variance panel examines the relationship between the fitted values and the squared Pearson residuals. A well‐fitted model should show a flat and horizontal reference line. Deviations from this horizontal line indicate heteroscedasticity. The collinearity panel presents the variance inflation factors (VIF) for each predictor variable. All our predictors (AGE, CRS, handfed, related, structure) have VIF values below the threshold, suggesting low collinearity and stable parameter estimates. The normality of random effects panel assesses the distribution of the random effects for the ANIMAL group. The theoretical quantiles of the random effects should align along the diagonal reference line if they are normally distributed.

After model selection, we applied a k‐folds cross validation. Our model including SSP, CRS, AGE, related, handfed and structure had a nearly perfect fit (*R* ^2^ = .992), considering also lower MAE and RMSE values (see Table 2). Goodness‐of‐fit diagnostics revealed no significant problems and indicated overall good model fit (see Figure 5). However, the variance was heteroscedastic, which is a common case in Poisson regression models.

**TABLE 2:** *K*‐folds cross‐validation results.

Mtry	RMSE	*R* ^2^	MAE
2	60.4779	.9990778	50.16869
4	58.9326	.9950437	49.84909
6	57.5201	.9923636	48.94835

*Note*: Root mean square error (RMSE) was used to select the optimal model using the smallest value. The final value used for the model was mtry = 6. *R* ^2^ indicates variance explained by the model. Lower MAE values indicate a model with better predictive accuracy.

**FIGURE 6:**
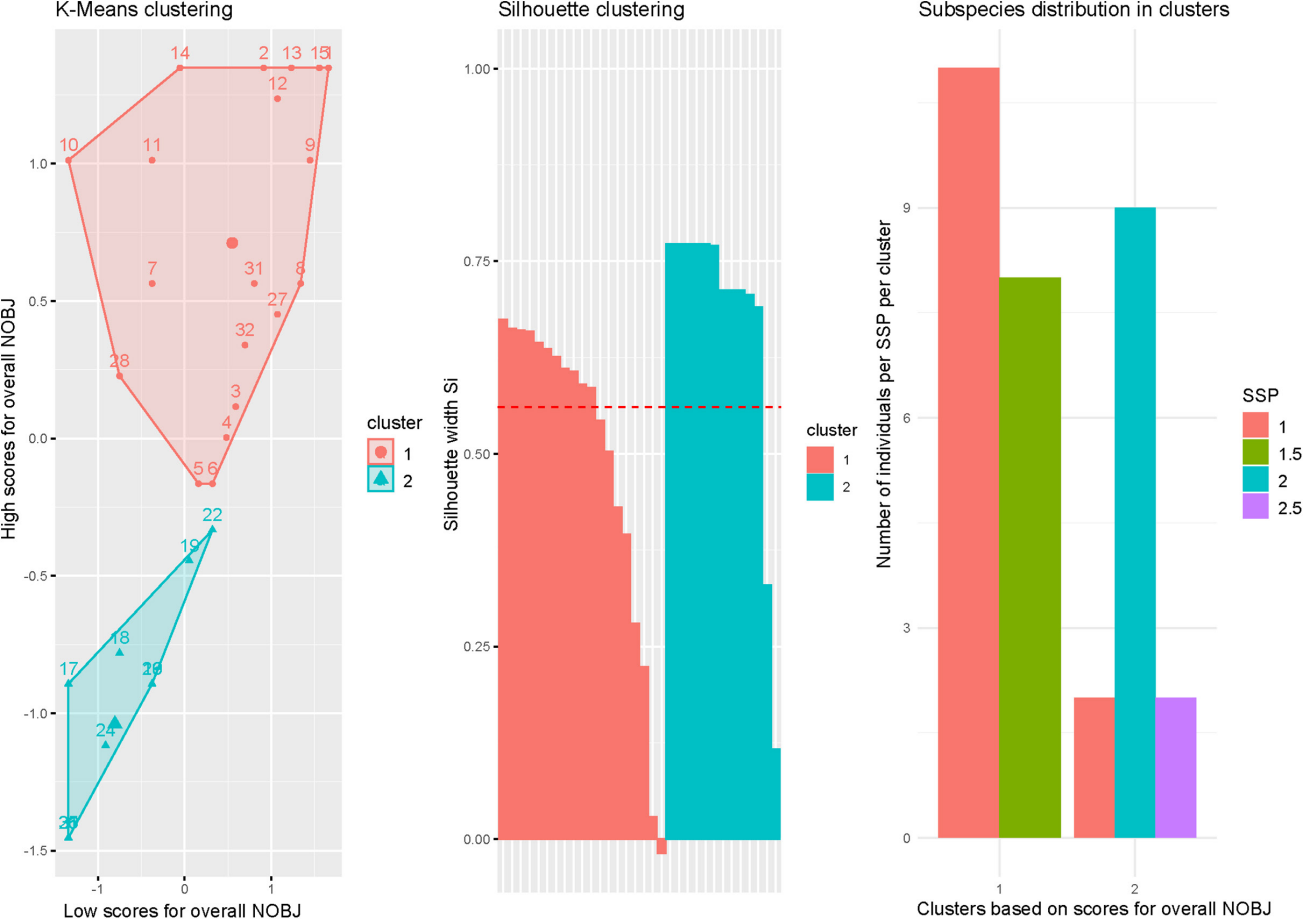
Silhouette plot of distance measures between all studied individuals, coloured by subspecies (red = *North American wolves*, green = *Canis lupus lycaon × Canis familiaris*, blue = *Canis lupus lupus*, pink = *Canis lupus lupus × Canis familiaris*), based on their frequency of bold behaviour.

**FIGURE A1:**
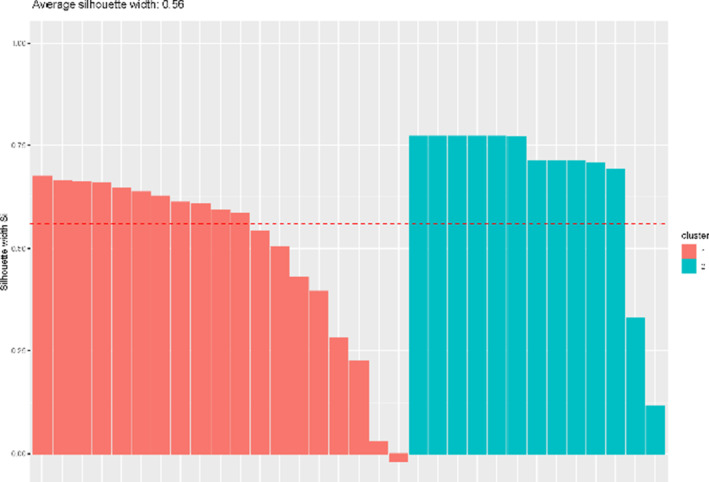
Average silhouette width of clusters.

To look at whether the respective individuals cluster based on their novel object interaction, we conducted a k‐means analysis. To assess the cohesion and separation of the formed clusters, an additional silhouette clustering was performed. The optimal number of clusters was determined to be 2, based on silhouette scores derived from k‐means analysis. A confusion matrix for model evaluation was generated to compare the predicted subspecies categories (derived from clustering) against the actual categories. Clustering revealed significant distinctions among subspecies (p≤.00001 $\left[\mathrm{Acc}>\mathrm{NIR}\right],\;\kappa =.73),$ with a confusion model accuracy of 0.8125. The average silhouette score from k‐fold validation was >0.58 (see Figure A1), indicating a solid separation between the clusters (see Figure 6).

**FIGURE A2:**
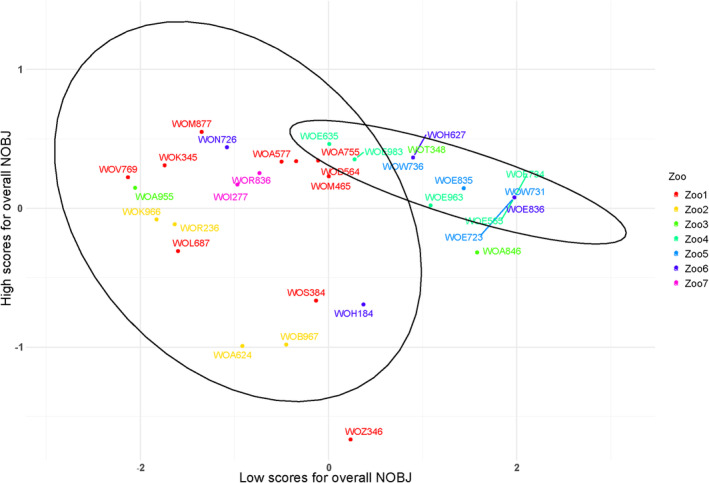
Individual animal's novel object interaction clustered by frequency, based on NMDS and silhouette clustering, clustered according to individual animal's NOBJ. Names represent individuals within the clusters. Colours represent the respective zoos. Clusters are indicated by ellipsis.

To address if clusters consisted of individuals primarily from different subspecies or zoos, we performed an NMDS based on frequency of NOBJ and personality traits associated with boldness. The groups were coloured according to subspecies and zoos. The figures show that the respective subspecies rather than zoos represent a population. The distance and spread between individuals in different zoos are diverse, whilst there is considerable overlap similar to the results of the GLMM and the cluster in subspecies (see Figure A2).

**FIGURE 7:**
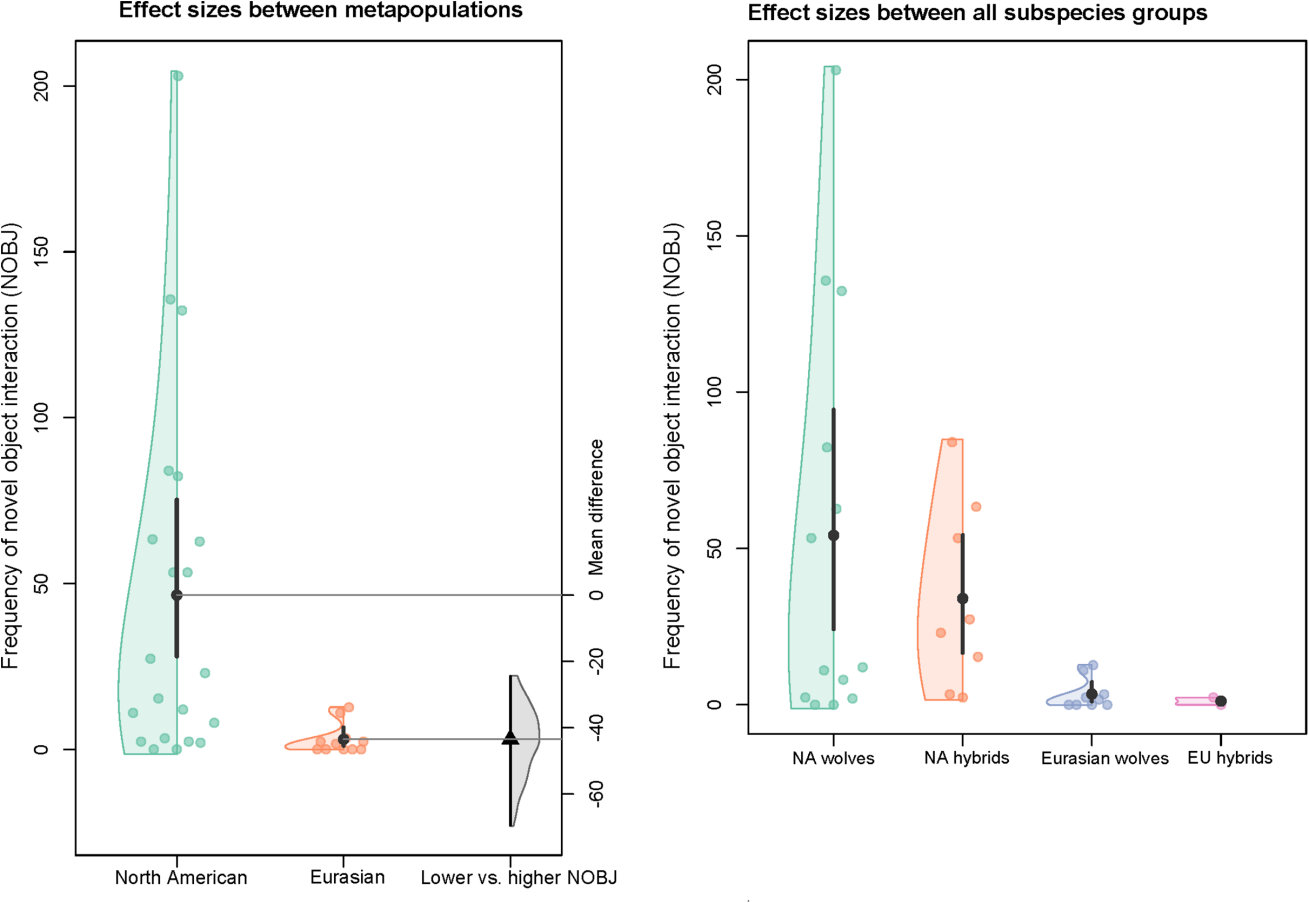
Differences in novel object interaction (NOBJ) between taxa and subspecies. Violin plots indicate density of NOBJ. Data points show respective NOBJ for each subspecies.

**FIGURE 8:**
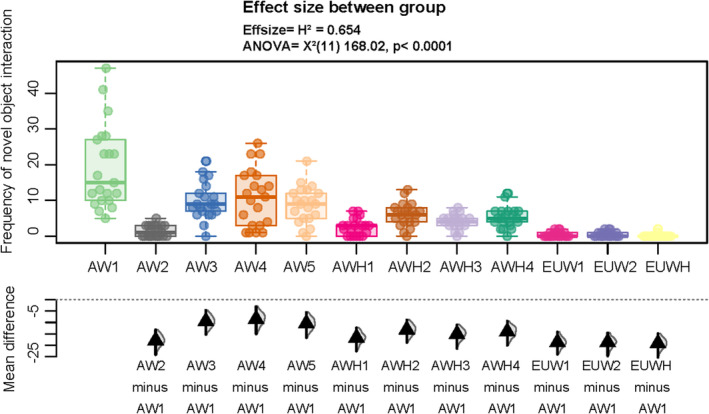
Between‐group differences in frequency of novel object interaction in American wolves (AW), American wolf‐dog hybrids (AWH), Eurasian wolves (EUW), and Eurasian wolf‐dog hybrids (EUWH). Boxplots represent medians (bar within the box), upper and lower quartiles (borders of box), lowest and highest cases within 1.5 times the IQR (bottom and top whiskers). Effect sizes with mean differences are shown under the boxplot.

We performed a non‐parametric ANOVA to test again for differences between the respective groups in the frequency of novel object interaction (see Figure 7). We discovered a highly significant difference between the American wolf groups and the Eurasian wolf groups (*X* ^2^(11) = 168.02; *d* = 0.654; p* =< .0001), with substantially lower inter‐subspecies differences (see Table A1; Figure 8). The hybrids between Eurasian wolves and domestic dogs also differed considerably from the timber wolf × dog hybrids (see Table A2).

### 3.2 Temporal stability of novel object interaction as an indicator of boldness

Differences in interindividual behaviour that are ‘repeatable over time and across situations’, sensu (Réale et al., 2007) can have fitness consequences, such as an increased individual mortality risk (Smith & Blumstein, 2008). Repeatable individual differences in behavioural responses to novelty, as a proxy for boldness, have been found in captive (Dammhahn & Almeling, 2012; Kanda et al., 2012) and wild (Bubac et al., 2018; López, 2020) mammals. To simulate to which extent the observed NOBJ would be constant over the experimental period, we modelled ARIMAs. ARIMAs are particularly suitable for short‐term forecasts, as they use the actual data of the observation period for their predictions. Therefore, we ran our forecast for a further 21 days, i.e. 42 days in total. As behaviour can adapt due to habituation or attrition, we ran two different forecast models to make predictions about the constancy of NOBJ. While our ARIMA models were adjusted for each group, our own forecast model used exponential smoothing methods with the same parameters for all groups. The results of our own model (AICc=90.15,BIC=92.37) are similar to those of the ARIMAs ($Q^{*}=1.6276,\mathrm{df}=3,\;p^{*}=.66$) and suggest that no significant decrease or increase in behaviour would be expected even with longer observation (see Table A3). We clustered the groups in our forecast plot based on the frequency of NOBJ in the forecast model to show overlaps between groups and respective subspecies. Variance was lower in groups with regular high frequency of NOBJ compared to those with a higher latency until NOBJ or irregular NOBJ. To check for a temporal change in NOBJ, we performed a segmented GLM with Model 1 plus variable ‘time’. Here we tested whether there were breaking points in the frequency of novel object interaction within the study period. Our segmented GLM revealed no estimated breakpoints ($p^{*}=.32$).

### 3.3 Differences in personality traits across the subspecies and groups

For each of the five personality factors, minimum and maximum values as well as means were evaluated for all animals included in the study (see Table 3).

**TABLE 3:** Values for personality factors for all involved animals.

Traits	Min	1st.Qu.	Mean ± SD	3rd.Qu.	Max
Curiosity	7.66	38.16	54.96 ± 25.33	79.72	92.85
Playfulness	9.52	45.69	53.29 ± 18.57	64.84	84.20
Extraversion	10.00	38.10	53.03 ± 21.13	71.25	85.00
Motivation	18.82	46.78	60.33 ± 20.37	18.82	46.78
Sociability	15.00	47.31	57.20 ± 14.97	67.02	79.99

**FIGURE 9:**
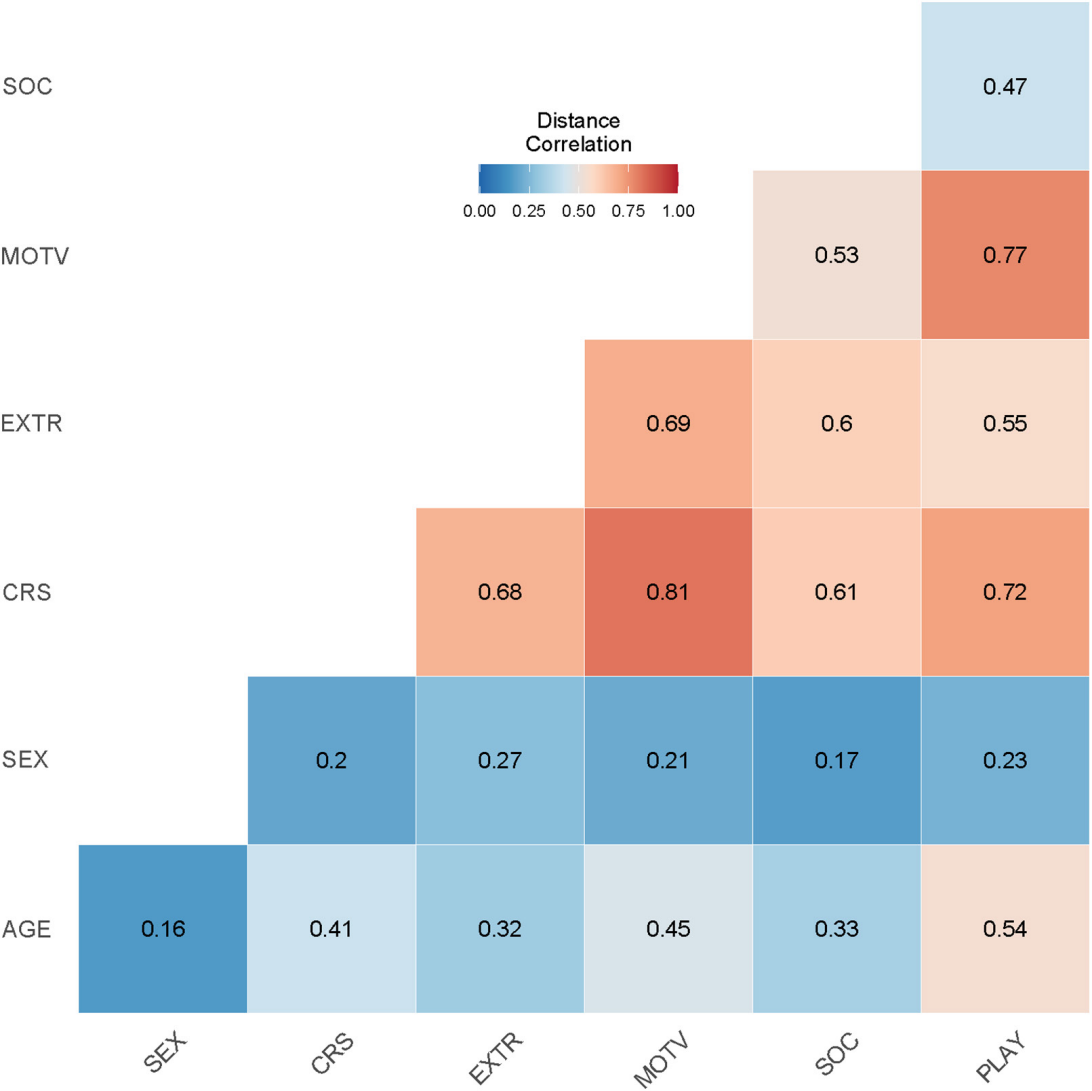
Correlation matrix of personality factors curiosity (CRS), playfulness (PLAY), motivation (MOTV), sociability (SOC), extraversion (EXTR), frequency of novel object interaction (NOBJ), and age. Positive correlations are indicated by red colour, negative correlations are indicated by blue colour. A higher degree of colour coverage indicates a stronger correlation.

Regarding personality traits, our non‐parametric ANOVA showed a significant difference between the timber wolf‐dog‐hybrids and the Eurasian wolf‐dog‐hybrids ($X^{2}\left(3\right)=15.24,p^{*}=.003$) in curiosity as well as in the traits motivation ($X^{2}\left(3\right)=8.53,p^{*}=.04$) and extraversion ($X^{2}\left(3\right)=12.97,p^{*}=.02$) between American wolves and Eurasian wolves, while American wolves and timber wolf‐dog hybrids as well as Eurasian wolves and Eurasian wolf‐dog‐hybrids did not significantly differ in their personality traits. As per distance correlation test (see Figure 9), there was a relevant positive correlation between curiosity (CRS) and playfulness (PLAY) ($R_{d}=.72,=p^{*}<.01$) as well as motivation (MOTV) ($R_{d}=.81,p=<.01$) and extraversion (EXTR) ($R_{d}=.61,p^{*}=<.01$). Extraversion was moderately positively correlated with PLAY ($R_{d}=.55,p^{*}=<.01$) and MOTV ($R_{d}=.69,p^{*}=<.01$). Sociability (SOC) was correlated moderately to weakly positively with EXTR ($R_{d}=.61,p^{*}=<0.01$), CRS ($R_{d}=.61,p^{*}=<.05$), MOTV ($R_{d}=.53,p^{*}=<.05$), and PLAY ($R_{d}=.47,p=.05$).

We accounted age‐based differences as well as differences in male and female individuals using generalised linear mixed effect models for each personality dimension. Age‐based differences were discovered for motivation and playfulness, showing that younger animals tend to score higher in these dimensions. In playfulness, age was the determining variable for differences, while for curiosity, motivation, and extraversion, subspecies explained more variation (see Table 4).

**TABLE 4:** Generalised linear mixed effect model results for subspecies (SSP), age‐ and sex‐based influences on each personality dimension.

Trait	Age *p*	Sex *p*
Curiosity	.03	.33
Playfulness	.0001	.04
Extraversion	.002	.13
Motivation	.10	.66
Sociability	.17	.12

We did not find any major differences between male and female wolves in their personality, but the frequency of novel object interaction varied between female and male wolves ($W=190,p^{*}=.01$).

## 4 DISCUSSION

Our results indicate that, in the wolf and hybrid groups studied, belonging to a certain taxon had the highest impact on novel object interaction and curiosity. Subspecies also had a greater influence on bold behaviour and personality than belonging to a specific group or the individual per se, although frequency of novel object interaction and curiosity were also influenced by the group in which the animals lived, suggesting that the behaviours could as well be influenced by the social community. Factors such as hand‐rearing hardly influenced the frequency of novel object interaction or curiosity. Sex did not influence the frequency of novel object interaction, likely due to the low sexual dimorphism in wolves. Group structure (pair versus pack) did not show a significant effect in our study, which may be biased by the fact that all Eurasian grey wolves lived in packs.

It is known that animal personality traits such as boldness or curiosity tend to be stable within life stages but not across (Cabrera et al., 2021). In line with findings on age‐related decline in boldness in butterflies (Kaiser et al., 2020) and domestic dogs (Starling et al., 2013), wolves, and hybrids tended to show more novel object interaction in young and middle age. Apart from playfulness, we could not find any significant effect of age on the examined personality dimensions. Considering that age only had a mild influence on bold behaviour according to our results and that the animals were neophilic or neophobic despite different ages, it can be assumed that life stage and experience were less relevant than other factors. The ARIMAs showed variance in the temporal stability of the NOBJ, with inter‐group variation. This result was expected as the groups varied in their frequency of NOBJ as well as in the temporal development of NOBJ to different degrees within and across groups. Species‐dependant developmental differences may be especially important for explaining behavioural disparities over ontogeny, especially if common explanatory factors such as age and gender have less effect. Our forecast model suggests that all groups would exhibit similar behaviours even after extended test intervals, indicating consistent and robust behaviour.

Since the animals all lived in similar conditions in zoological institutions and were acquired or reared under comparable conditions, it is reasonable to assume that their species ecology has a role in behaviour, as does species history, even in groups no longer affected by natural environmental influences found in the wild. Strong evolutionary drivers are known to be conserved and can therefore be influential across multiple generations or substantially affect future populations in the case of high adaptation necessities (Garroway & Sheldon, 2013; Langkilde et al., 2017).

Factors that influence exposure risk, such as the availability of refuge habitats, are also influenced by human activities and landscape‐level impacts (Iliopoulos et al., 2014; Jędrzejewski et al., 2004; Llaneza et al., 2012; Thurber et al., 1994). Wolves commonly tend to avoid areas with high human densities due to increased risk of anthropogenic mortality (Gurarie et al., 2011; Murray et al., 2010; Ordiz et al., 2015; Theuerkauf, 2009; Whittington et al., 2005; Zimmermann et al., 2014). In Europe, wolves commonly travel through forested areas and avoid anthropogenetic settlements as much as possible (Carricondo‐Sanchez et al., 2020; Kaartinen et al., 2005); another example is Yellowstone National Park, where wolves adapted their use of roads for travelling in order to avoid vehicles, since the mortality rate of wolves has increased due to car accidents (Anton et al., 2020), a mechanism related to non‐genetic inheritance of behaviour (see Reddon et al., 2012). On the other hand, in arctic regions or similar areas where wolves have little contact with human‐based threats (Mech, 1988), these naïve wolves often show stronger approach behaviour to potential risks such as human camps, people, or unfamiliar objects introduced into their habitat by humans (Marquard‐Petersen, 2022; Miller, 1995; Parmelee, 1964; Pruitt, 1973). In wolves that have not experienced hunting or dispersal, curious behaviour, and approach is sometimes even part of their normal behaviour, for example in Canis lupus arctos (Marquard‐Petersen, 2022; Mech, 1988). Since repeated interactions between anthropogenic influences, wolf behaviour and consequences of these shape reaction norms of wolf populations, gene–environment interactions (reaction norms) are as well likely to play a role for subspecies‐related behaviours (see Dingemanse et al., 2003; Réale et al., 2007).

Considering that curiosity is strongly associated with exploratory tendencies and with boldness, the link between the trait curiosity (CRS) and the frequency of novel object interaction is expected. As personality traits, and in particular the boldness‐related traits, are likely to play a role in population response to anthropogenic disturbance (Merrick & Koprowski, 2017), certain behavioural types may be more or less susceptible to the effects of human activities (Smith & Blumstein, 2008).

It is known that animals show experience‐based adaptability in their behaviour (Magnhagen & Staffan, 2005). In rainbow trout (*Oncorhynchus mykiss*), bold individuals observing shy leaders became more timid, as evidenced by increased latency to approach a novel object, while shy observers observing bold leaders remained cautious and did not change their responses to novelty (Frost et al., 2007). Breeding experiments in rainbow trout (Fevolden et al., 1999) and free‐living bird populations (Béziers et al., 2019; Jenkins et al., 2014; Stedman et al., 2017) showed higher heritability for stress‐related neurobiological response mechanisms (see Taborsky et al., 2020). A phylogenetically controlled comparative analysis in tetrapods also indicates that stress responses in species that are exposed to frequent challenges underlie evolutionary mechanisms and are consequently adapted. This proposes that both social and evolutive mechanisms play a role in the shaping and manifestation of behavioural profiles such as boldness‐shyness.

Taking into account that wolves are highly social animals that cooperate and learn intensively from each other, mechanisms of social feedback and social transmission, e.g. positive feedbacks between individual state and behaviour (see Sih et al., 2015), are also likely to be of significance. Social transmission of information is important for the fitness of individuals within populations, as it allows them to obtain useful information simply by observing each other. Such mechanisms reduce the costs associated with first‐hand learning (Brown & Laland, 2003). Wolves decide and behave based on early life experiences (Karlin & Chadwick, 2012; Pilot et al., 2006), as do coyotes (*Canis latrans*) (Sacks et al., 2008). It is generally accepted that the transmission of habitat experiences is crucial for offspring in wolves (van Liere et al., 2021). As a result of parental education, the new generation would choose a habitat comparable to the one in which it was raised to survive efficiently (Davis & Stamps, 2004; Laland, 2004; Packard, 2012, 2019; van Liere et al., 2021), although it is known that wolves behave more boldly after dispersal than in their original environment (Barry et al., 2020).

In many species, boldness leads to both higher risks and higher rewards (Cote et al., 2010). For example, bold swift foxes (*Vulpes velox*) showed lower survival rates than their shyer conspecifics in a reintroduction programme (Bremner‐Harrison et al., 2004). Parasitic infections, such as *Toxoplasma gondii* infection, can increase boldness and risk‐taking in wild animals (Meyer et al., 2022) and should be considered when addressing variation in boldness of wild populations. However, infection risk is low in captive animals. Mid‐ or long‐term influences that lead to population‐based variation might be more likely, especially if variation is population‐specific among different, unrelated groups. We, therefore, suggest that differences in boldness and related traits in different wolf subspecies could be influenced by variation in risk‐avoiding traits over a long period of time of the respective subspecies and are linked to the conditions and challenges of their habitat as well as associated survival and dispersal decisions. We conjecture that both genetic adaptations, evolutive factors and social learning affect the subspecies‐related differences in boldness and associated personality traits in distinct wolf subspecies, even in captive animals. Assessment of personality traits such as boldness requires repeated measurements on the same individuals over a longer period of time. First, animals may become habituated to a test if they experience it repeatedly (Biro & Stamps, 2015), although our data indicate no habituation effect in our trial. Second, test–retest intervals may also affect behavioural outcomes, as animals are more likely to exhibit consistent behaviour over test intervals that are shorter relative to the animals' lifespan (Bell, 2007). Additionally, single assays of differences in bold behaviours might not be representative for bold behaviour in other contexts (Beckmann & Biro, 2013). Our results indicate differences in boldness between wolf populations related to species ecology and species history, but further investigations within additional contexts, larger sample sizes and comparisons with wild populations are necessary to control our findings.

## AUTHOR CONTRIBUTIONS

**Hana Tebelmann:** Conceptualization (lead); data curation (lead); formal analysis (lead); investigation (lead); methodology (equal); project administration (equal); validation (lead); visualization (lead); writing – original draft (lead); writing – review and editing (equal). **Udo Ganslosser:** Conceptualization (supporting); methodology (supporting); project administration (supporting); resources (equal); supervision (lead); writing – review and editing (supporting).

## FUNDING INFORMATION

This research received internal support from the MAMMALIA working group, Institute for Zoology and Evolutionary Research, Friedrich Schiller Universität Jena.

## CONFLICT OF INTEREST STATEMENT

The authors declare no conflict of interest.

## Supporting information


Table S1

## Data Availability

Data pertinent to this study are provided in the file WolfData available via Zenodo under https://zenodo.org/records/13294107, with specific details regarding ID and group affiliations of the animals anonymised in accordance with agreements with participating zoological facilities. Table S1 offering further insights into the data is accessible via Zenodo under 10.5281/zenodo.10949588.

## APPENDIX 1

**TABLE A1:** Pseudonymised list including all animals included in the study.

Animal_ID	Subspecies	Age	Sex	Facility	Group
WOV769	1	1.5	Female	ZOO_1	AW1
WOL687	1.1	2.5	Male	ZOO_1	AW1
WOM465	1.1	4	Male	ZOO_1	AW2
WOA755	1.1	12	Female	ZOO_1	AW2
WOK345	1	9	Female	ZOO_1	AW3
WOZ346	1	11	Male	ZOO_1	AW3
WOB967	1.1	5	Male	ZOO_2	AW4
WOR236	1.1	11	Female	ZOO_2	AW4
WOK966	1.1	4	Male	ZOO_2	AW4
WOA624	1.1	6	Male	ZOO_2	AW4
WOA955	1.1	4.5	Female	ZOO_3	AW5
WOT348	1	4.5	Male	ZOO_3	AW5
WOA846	1	1.5	Male	ZOO_3	AW5
WOA577	1.5	1	Male	ZOO_1	AWH1
WOD564	1.5	3	Female	ZOO_1	AWH1
WOS384	1.5	9	Male	ZOO_1	AWH2
WOM877	1.5	5	Female	ZOO_1	AWH2
WOH184	1.5	5	Female	ZOO_6	AWH3
WOH627	2.5	4	Male	ZOO_6	AWH3
WOR836	1.5	5	Male	ZOO_7	AWH4
WOV769	1.5	6	Female	ZOO_7	AWH4
WOE963	2	3	Female	ZOO_4	EU1
WOE983	2	3	Male	ZOO_4	EU1
WOE565	2	8	Female	ZOO_4	EU1
WOE734	2	7	Male	ZOO_4	EU1
WOE635	2	4	Female	ZOO_4	EU1
WOW736	2	10	Male	ZOO_5	EU2
WOE835	2	10	Male	ZOO_5	EU2
WOE723	2	10	Female	ZOO_5	EU2
WOW731	2	8	Male	ZOO_5	EU2
WON726	1.5	10	Female	ZOO_5	EU2
WOE836	2.5	4	Male	ZOO_6	EUWH
WOI277	1.5	4	Female	ZOO_6	EUWH

Abbreviations: AW, North American wolves; AWH, North American wolf hybrids; EUW, Eurasian wolves; EUWH, Eurasian wolf hybrids.

## APPENDIX 2

**TABLE A2:** Estimated differences between the included groups.

Group		*p*‐Value
AW1	AW2	<.00001
AW1	AW3	.031
AW1	AW4	.444
AW1	AW5	.033
AW2	AW3	<.00001
AW2	AW4	<.0001
AW2	AW5	<.00001
AW3	AW4	.601
AW3	AW5	.509
AW4	AW5	.9
AWH1	AWH2	.005
AWH1	AWH3	.183
AWH1	AWH4	.04
AWH2	AWH3	.0002
AWH2	AWH4	.292
AWH3	AWH4	.332
EUWH	EU1	.03
EUWH	EU2	.01
EU1	EU2	.611
EU1	AW1	<.00001
EU1	AW2	.508
EU1	AW3	<.00001
EU1	AW4	<.00001
EU1	AW5	<.00001
EU1	AWH1	.0329
EU1	AWH2	<.00001
EU1	AWH3	<.00001
EU1	AWH4	<.00001
EU2	AW1	<.00001
EU2	AW2	.822
EU2	AW3	<.0001
EU2	AW4	<.00001
EU2	AW5	<.0001
EU2	AWH1	.638
EU2	AWH2	.9
EU2	AWH3	<.0001
EU2	AWH4	<.0001
EUWH	AW1	<.00001
EUWH	AW2	.116
EUWH	AW3	<.00001
EUWH	AW4	<.00001
EUWH	AW5	<.00001
EUWH	AWH1	.0002
EUWH	AWH2	<.00001
EUWH	AWH3	<.00001
EUWH	AWH4	<.00001

## APPENDIX 4

**TABLE A3:** ARIMA model output for all tested groups.

Group: Group1
Series: group_data
ARIMA (0,0,0) with non‐zero mean
Coefficients: mean = 19.2857; SE = 2.4846
Sigma^2^ = 136.1: log likelihood = −80.88
AIC = 165.75; AICc = 166.42; BIC = 167.84

Point	Forecast	Lo 80	Hi 80	Lo 95	Hi 95
22	19.28571	4.334105	34.23732	−3.580799	42.15223
23	19.28571	4.334105	34.23732	−3.580799	42.15223
24	19.28571	4.334105	34.23732	−3.580799	42.15223
25	19.28571	4.334105	34.23732	−3.580799	42.15223
26	19.28571	4.334105	34.23732	−3.580799	42.15223
27	19.28571	4.334105	34.23732	−3.580799	42.15223
28	19.28571	4.334105	34.23732	−3.580799	42.15223
29	19.28571	4.334105	34.23732	−3.580799	42.15223
30	19.28571	4.334105	34.23732	−3.580799	42.15223
31	19.28571	4.334105	34.23732	−3.580799	42.15223
32	19.28571	4.334105	34.23732	−3.580799	42.15223
33	19.28571	4.334105	34.23732	−3.580799	42.15223
34	19.28571	4.334105	34.23732	−3.580799	42.15223
35	19.28571	4.334105	34.23732	−3.580799	42.15223
36	19.28571	4.334105	34.23732	−3.580799	42.15223
37	19.28571	4.334105	34.23732	−3.580799	42.15223
38	19.28571	4.334105	34.23732	−3.580799	42.15223
39	19.28571	4.334105	34.23732	−3.580799	42.15223
40	19.28571	4.334105	34.23732	−3.580799	42.15223
41	19.28571	4.334105	34.23732	−3.580799	42.15223
42	19.28571	4.334105	34.23732	−3.580799	42.15223

Group: Group2
Series: group_data
ARIMA (0,0,1) with non‐zero mean
Coefficients: ma1 mean = 0.4981 1.3868; SE = 0.2323 0.4556
Sigma^2^ = 2.212: log likelihood = −37.22
AIC = 80.45; AICc = 81.86; BIC = 83.58

Point	Forecast	Lo 80	Hi 80	Lo 95	Hi 95
22	0.917548	–0.9883999	2.823496	–1.997348	3.832444
23	1.386777	–0.7424856	3.516040	–1.869649	4.643204
24	1.386777	–0.7424856	3.516040	–1.869649	4.643204
25	1.386777	–0.7424856	3.516040	–1.869649	4.643204
26	1.386777	–0.7424856	3.516040	–1.869649	4.643204
27	1.386777	–0.7424856	3.516040	–1.869649	4.643204
28	1.386777	–0.7424856	3.516040	–1.869649	4.643204
29	1.386777	–0.7424856	3.516040	–1.869649	4.643204
30	1.386777	–0.7424856	3.516040	–1.869649	4.643204
31	1.386777	–0.7424856	3.516040	–1.869649	4.643204
32	1.386777	–0.7424856	3.516040	–1.869649	4.643204
33	1.386777	–0.7424856	3.516040	–1.869649	4.643204
34	1.386777	–0.7424856	3.516040	–1.869649	4.643204
35	1.386777	–0.7424856	3.516040	–1.869649	4.643204
36	1.386777	–0.7424856	3.516040	–1.869649	4.643204
37	1.386777	–0.7424856	3.516040	–1.869649	4.643204
38	1.386777	–0.7424856	3.516040	–1.869649	4.643204
39	1.386777	–0.7424856	3.516040	–1.869649	4.643204
40	1.386777	–0.7424856	3.516040	–1.869649	4.643204
41	1.386777	–0.7424856	3.516040	–1.869649	4.643204
42	1.386777	–0.7424856	3.516040	–1.869649	4.643204

Group: Group3
Series: group_data
ARIMA (0,0,1) with non‐zero mean
Coefficients: ma1 mean = 0.5228 9.5573; SE = 0.1526 1.4139
Sigma^2^ = 20.6: log likelihood = −60.67
AIC = 127.34; AICc = 128.75; BIC = 130.48

Point	Forecast	Lo 80	Hi 80	Lo 95	Hi 95
22	5.757412	–0.05886908	11.57369	−3.1378224	14.65265
23	9.557341	2.99420955	16.12047	−0.4801024	19.59478
24	9.557341	2.99420955	16.12047	−0.4801024	19.59478
25	9.557341	2.99420955	16.12047	−0.4801024	19.59478
26	9.557341	2.99420955	16.12047	−0.4801024	19.59478
27	9.557341	2.99420955	16.12047	−0.4801024	19.59478
28	9.557341	2.99420955	16.12047	−0.4801024	19.59478
29	9.557341	2.99420955	16.12047	−0.4801024	19.59478
30	9.557341	2.99420955	16.12047	−0.4801024	19.59478
31	9.557341	2.99420955	16.12047	−0.4801024	19.59478
32	9.557341	2.99420955	16.12047	−0.4801024	19.59478
33	9.557341	2.99420955	16.12047	−0.4801024	19.59478
34	9.557341	2.99420955	16.12047	−0.4801024	19.59478
35	9.557341	2.99420955	16.12047	−0.4801024	19.59478
36	9.557341	2.99420955	16.12047	−0.4801024	19.59478
37	9.557341	2.99420955	16.12047	−0.4801024	19.59478
38	9.557341	2.99420955	16.12047	−0.4801024	19.59478
39	9.557341	2.99420955	16.12047	−0.4801024	19.59478
40	9.557341	2.99420955	16.12047	−0.4801024	19.59478
41	9.557341	2.99420955	16.12047	−0.4801024	19.59478
42	9.557341	2.99420955	16.12047	−0.4801024	19.59478

Group: Group4
Series: group_dat
ARIMA (0,0,0) with non‐zero mean
Coefficients: mean = 10.7619; SE = 1.6955
Sigma^2^ = 63.39: log likelihood = −72.85
AIC = 149.71; AICc = 150.37; BIC = 151.8

Point	Forecast	Lo 80	Hi 80	Lo 95	Hi 95
22	10.7619	0.55843	20.96538	−4.842963	26.36677
23	10.7619	0.55843	20.96538	−4.842963	26.36677
24	10.7619	0.55843	20.96538	−4.842963	26.36677
25	10.7619	0.55843	20.96538	−4.842963	26.36677
26	10.7619	0.55843	20.96538	−4.842963	26.36677
27	10.7619	0.55843	20.96538	−4.842963	26.36677
28	10.7619	0.55843	20.96538	−4.842963	26.36677
29	10.7619	0.55843	20.96538	−4.842963	26.36677
30	10.7619	0.55843	20.96538	−4.842963	26.36677
31	10.7619	0.55843	20.96538	−4.842963	26.36677
32	10.7619	0.55843	20.96538	−4.842963	26.36677
33	10.7619	0.55843	20.96538	−4.842963	26.36677
34	10.7619	0.55843	20.96538	−4.842963	26.36677
35	10.7619	0.55843	20.96538	−4.842963	26.36677
36	10.7619	0.55843	20.96538	−4.842963	26.36677
37	10.7619	0.55843	20.96538	−4.842963	26.36677
38	10.7619	0.55843	20.96538	−4.842963	26.36677
39	10.7619	0.55843	20.96538	−4.842963	26.36677
40	10.7619	0.55843	20.96538	−4.842963	26.36677
41	10.7619	0.55843	20.96538	−4.842963	26.36677
42	10.7619	0.55843	20.96538	−4.842963	26.36677

Group: Group5
Series: group_data
ARIMA (1,0,0) with non‐zero mean
Coefficients: ar1 mean = 0.5010 8.3895; SE = 0.1941 1.8429
Sigma^2^ = 20.81: log likelihood = −60.76
AIC = 127.52; AICc = 128.94; BIC = 130.66

Point	Forecast	Lo 80	Hi 80	Lo 95	Hi 95
22	6.691255	0.845421	12.53709	−2.249176	15.63169
23	7.538616	1.000093	14.07714	−2.461193	17.53843
24	7.963166	1.261992	14.66434	−2.285395	18.21173
25	8.175876	1.434489	14.91726	−2.134185	18.48594
26	8.282449	1.531005	15.03389	−2.042993	18.60789
27	8.335845	1.581879	15.08981	−1.993455	18.66514
28	8.362598	1.607999	15.11720	−1.967670	18.69287
29	8.376001	1.621244	15.13076	−1.954509	18.70651
30	8.382717	1.627919	15.13751	−1.947855	18.71329
31	8.386082	1.631274	15.14089	−1.944505	18.71667
32	8.387768	1.632957	15.14258	−1.942823	18.71836
33	8.388612	1.633801	15.14342	−1.941980	18.71920
34	8.389035	1.634224	15.14385	−1.941557	18.71963
35	8.389247	1.634436	15.14406	−1.941345	18.71984
36	8.389354	1.634543	15.14416	−1.941238	18.71995
37	8.389407	1.634596	15.14422	−1.941185	18.72000
38	8.389434	1.634622	15.14424	−1.941159	18.72003
39	8.389447	1.634636	15.14426	−1.941145	18.72004
40	8.389454	1.634643	15.14426	−1.941138	18.72005
41	8.389457	1.634646	15.14427	−1.941135	18.72005
42	8.389459	1.634648	15.14427	−1.941133	18.72005

Group: Group6
Series: group_data
ARIMA (1,0,0) with non‐zero mean
Coefficients: ar1 mean = 0.4685 2.4549; SE = 0.1946 0.7951
Sigma^2^ = 4.393: log likelihood = −44.41
AIC = 94.82 AICc = 96.23 BIC = 97.96

Point	Forecast	Lo 80	Hi 80	Lo 95	Hi 95
22	1.304702	−1.3813658	3.990771	−2.803284	5.412689
23	1.916000	−1.0502808	4.882280	−2.620535	6.452534
24	2.202413	−0.8219073	5.226734	−2.422886	6.827712
25	2.336608	−0.7003056	5.373521	−2.307950	6.981166
26	2.399482	−0.6401883	5.439153	−2.249293	7.048258
27	2.428941	−0.6113344	5.469217	−2.220759	7.078642
28	2.442744	−0.5976647	5.483152	−2.207160	7.092647
29	2.449211	−0.5912269	5.489648	−2.200737	7.099159
30	2.452241	−0.5882034	5.492685	−2.197717	7.102199
31	2.453660	−0.5867851	5.494106	−2.196300	7.103620
32	2.454325	−0.5861203	5.494771	−2.195635	7.104286
33	2.454637	−0.5858087	5.495083	−2.195323	7.104598
34	2.454783	−0.5856627	5.495229	−2.195177	7.104744
35	2.454852	−0.5855943	5.495297	−2.195109	7.104812
36	2.454884	−0.5855622	5.495329	−2.195077	7.104844
37	2.454899	−0.5855472	5.495344	−2.195062	7.104859
38	2.454906	−0.5855402	5.495351	−2.195055	7.104866
39	2.454909	−0.5855369	5.495355	−2.195052	7.104870
40	2.454910	−0.5855353	5.495356	−2.195050	7.104871
41	2.454911	−0.5855346	5.495357	−2.195049	7.104872
42	2.454912	−0.5855343	5.495357	−2.195049	7.104872

Group: Group7
Series: group_data
ARIMA (1,0,0) with non‐zero mean
Coefficients: ar1 mean = 0.4902 5.6257; SE = 0.2235 1.2389
Sigma^2^ = 9.129: log likelihood = −52.11
AIC = 110.21 AICc = 111.62 BIC = 113.34

Point	Forecast	Lo 80	Hi 80	Lo 95	Hi 95
22	2.867702	−1.00450355	6.739907	−3.054325	8.789728
23	4.273581	−0.03891723	8.586080	−2.321816	10.868979
24	4.962808	0.55103391	9.374583	−1.784418	11.710035
25	5.300699	0.86539594	9.736003	−1.482512	12.083911
26	5.466349	1.02540930	9.907289	−1.325482	12.258181
27	5.547558	1.10526486	9.989852	−1.246343	12.341460
28	5.587371	1.14475207	10.029990	−1.207028	12.381770
29	5.606889	1.16419181	10.049586	−1.187630	12.401407
30	5.616457	1.17374161	10.059173	−1.178090	12.411005
31	5.621148	1.17842805	10.063868	−1.173406	12.415702
32	5.623448	1.18072669	10.066169	−1.171108	12.418004
33	5.624575	1.18185387	10.067297	−1.169981	12.419132
34	5.625128	1.18240652	10.067850	−1.169428	12.419685
35	5.625399	1.18267747	10.068121	−1.169157	12.419956
36	5.625532	1.18281031	10.068254	−1.169024	12.420088
37	5.625597	1.18287544	10.068319	−1.168959	12.420153
38	5.625629	1.18290736	10.068351	−1.168927	12.420185
39	5.625645	1.18292302	10.068366	−1.168912	12.420201
40	5.625652	1.18293069	10.068374	−1.168904	12.420209
41	5.625656	1.18293445	10.068378	−1.168900	12.420213
42	5.625658	1.18293630	10.068380	−1.168898	12.420214

Group: Group8
Series: group_data
ARIMA (0,1,0)
Sigma^2^ = 2.25: log likelihood = −36.49
AIC = 74.98; AICc = 75.2; BIC = 75.97

Point	Forecast	Lo 80	Hi 80	Lo 95	Hi 95
22	3	1.0776713	4.922329	0.06005193	5.939948
23	3	0.2814167	5.718583	−1.15771443	7.157714
24	3	−0.3295710	6.329571	−2.09213942	8.092139
25	3	−0.8446574	6.844657	−2.87989613	8.879896
26	3	−1.2984577	7.298458	−3.57392373	9.573924
27	3	−1.7087245	7.708724	−4.20137264	10.201373
28	3	−2.0860037	8.086004	−4.77837145	10.778371
29	3	−2.4371667	8.437167	−5.31542886	11.315429
30	3	−2.7669861	8.766986	−5.81984420	11.819844
31	3	−3.0789372	9.078937	−6.29693210	12.296932
32	3	−3.3756431	9.375643	−6.75070464	12.750705
33	3	−3.6591420	9.659142	−7.18427885	13.184279
34	3	−3.9310548	9.931055	−7.60013350	13.600134
35	3	−4.1926954	10.192695	−8.00027840	14.000278
36	3	−4.4451471	10.445147	−8.38636990	14.386370
37	3	−4.6893149	10.689315	−8.75979227	14.759792
38	3	−4.9259643	10.925964	−9.12171642	15.121716
39	3	−5.1557500	11.155750	−9.47314329	15.473143
40	3	−5.3792366	11.379237	−9.81493653	15.814937
41	3	−5.5969154	11.596915	−10.14784746	16.147847
42	3	−5.8092168	11.809217	−10.47253456	16.472535

Group: Group9
Series: group_data
ARIMA (0,0,0) with non‐zero mean
Coefficients: mean = 5.2381; SE = 0.6132
Sigma^2^ = 8.29: log likelihood = −51.49
AIC = 106.99; AICc = 107.65; BIC = 109.08

Point	Forecast	Lo 80	Hi 80	Lo 95	Hi 95
22	5.238095	1.5481	8.928091	−0.4052657	10.88146
23	5.238095	1.5481	8.928091	−0.4052657	10.88146
24	5.238095	1.5481	8.928091	−0.4052657	10.88146
25	5.238095	1.5481	8.928091	−0.4052657	10.88146
26	5.238095	1.5481	8.928091	−0.4052657	10.88146
27	5.238095	1.5481	8.928091	−0.4052657	10.88146
28	5.238095	1.5481	8.928091	−0.4052657	10.88146
29	5.238095	1.5481	8.928091	−0.4052657	10.88146
30	5.238095	1.5481	8.928091	−0.4052657	10.88146
31	5.238095	1.5481	8.928091	−0.4052657	10.88146
32	5.238095	1.5481	8.928091	−0.4052657	10.88146
33	5.238095	1.5481	8.928091	−0.4052657	10.88146
34	5.238095	1.5481	8.928091	−0.4052657	10.88146
35	5.238095	1.5481	8.928091	−0.4052657	10.88146
36	5.238095	1.5481	8.928091	−0.4052657	10.88146
37	5.238095	1.5481	8.928091	−0.4052657	10.88146
38	5.238095	1.5481	8.928091	−0.4052657	10.88146
39	5.238095	1.5481	8.928091	−0.4052657	10.88146
40	5.238095	1.5481	8.928091	−0.4052657	10.88146
41	5.238095	1.5481	8.928091	−0.4052657	10.88146
42	5.238095	1.5481	8.928091	−0.4052657	10.88146

Group: Group10
Series: group_data
ARIMA (0,0,0) with zero mean
Sigma^2^ = 0.2381: log likelihood = −14.73
AIC = 31.46; AICc = 31.67; BIC = 32.5

Point	Forecast	Lo 80	Hi 80	Lo 95	Hi 95
22	0	−0.6253331	0.6253331	−0.9563645	0.9563645
23	0	−0.6253331	0.6253331	−0.9563645	0.9563645
24	0	−0.6253331	0.6253331	−0.9563645	0.9563645
25	0	−0.6253331	0.6253331	−0.9563645	0.9563645
26	0	−0.6253331	0.6253331	−0.9563645	0.9563645
27	0	−0.6253331	0.6253331	−0.9563645	0.9563645
28	0	−0.6253331	0.6253331	−0.9563645	0.9563645
29	0	−0.6253331	0.6253331	−0.9563645	0.9563645
30	0	−0.6253331	0.6253331	−0.9563645	0.9563645
31	0	−0.6253331	0.6253331	−0.9563645	0.9563645
32	0	−0.6253331	0.6253331	−0.9563645	0.9563645
33	0	−0.6253331	0.6253331	−0.9563645	0.9563645
34	0	−0.6253331	0.6253331	−0.9563645	0.9563645
35	0	−0.6253331	0.6253331	−0.9563645	0.9563645
36	0	−0.6253331	0.6253331	−0.9563645	0.9563645
37	0	−0.6253331	0.6253331	−0.9563645	0.9563645
38	0	−0.6253331	0.6253331	−0.9563645	0.9563645
39	0	−0.6253331	0.6253331	−0.9563645	0.9563645
40	0	−0.6253331	0.6253331	−0.9563645	0.9563645
41	0	−0.6253331	0.6253331	−0.9563645	0.9563645
42	0	−0.6253331	0.6253331	−0.9563645	0.9563645

Group: Group11
Series: group_data
ARIMA (0,0,0) with non‐zero mean
Coefficients: mean = 0.5714; SE = 0.1726
Sigma^2^ = 0.6571: log likelihood = −24.88
AIC = 53.75; AICc = 54.42; BIC = 55.84

Point	Forecast	Lo 80	Hi 80	Lo 95	Hi 95
22	0.5714286	−0.4674529	1.61031	−1.017403	2.160261
23	0.5714286	−0.4674529	1.61031	−1.017403	2.160261
24	0.5714286	−0.4674529	1.61031	−1.017403	2.160261
25	0.5714286	−0.4674529	1.61031	−1.017403	2.160261
26	0.5714286	−0.4674529	1.61031	−1.017403	2.160261
27	0.5714286	−0.4674529	1.61031	−1.017403	2.160261
28	0.5714286	−0.4674529	1.61031	−1.017403	2.160261
29	0.5714286	−0.4674529	1.61031	−1.017403	2.160261
30	0.5714286	−0.4674529	1.61031	−1.017403	2.160261
31	0.5714286	−0.4674529	1.61031	−1.017403	2.160261
32	0.5714286	−0.4674529	1.61031	−1.017403	2.160261
33	0.5714286	−0.4674529	1.61031	−1.017403	2.160261
34	0.5714286	−0.4674529	1.61031	−1.017403	2.160261
35	0.5714286	−0.4674529	1.61031	−1.017403	2.160261
36	0.5714286	−0.4674529	1.61031	−1.017403	2.160261
37	0.5714286	−0.4674529	1.61031	−1.017403	2.160261
38	0.5714286	−0.4674529	1.61031	−1.017403	2.160261
39	0.5714286	−0.4674529	1.61031	−1.017403	2.160261
40	0.5714286	−0.4674529	1.61031	−1.017403	2.160261
41	0.5714286	−0.4674529	1.61031	−1.017403	2.160261
42	0.5714286	−0.4674529	1.61031	−1.017403	2.160261

Group: Group12
Series: group_data
ARIMA (1,0,0) with zero mean
Coefficients: ar1 = 0.7084. SE = 0.1385
Sigma^2^ = 0.5228: log likelihood = −22.82
AIC = 49.65; AICc = 50.32; BIC = 51.74

Point	Forecast	Lo 80	Hi 80	Lo 95	Hi 95
22	0	−0.926650	0.926650	−1.417189	1.417189
23	0	−1.135576	1.135576	−1.736714	1.736714
24	0	−1.227077	1.227077	−1.876653	1.876653
25	0	−1.270509	1.270509	−1.943076	1.943076
26	0	−1.291751	1.291751	−1.975563	1.975563
27	0	−1.302279	1.302279	−1.991664	1.991664
28	0	−1.307530	1.307530	−1.999694	1.999694
29	0	−1.310156	1.310156	−2.003711	2.003711
30	0	−1.311472	1.311472	−2.005724	2.005724
31	0	−1.312132	1.312132	−2.006733	2.006733
32	0	−1.312463	1.312463	−2.007239	2.007239
33	0	−1.312629	1.312629	−2.007493	2.007493
34	0	−1.312712	1.312712	−2.007620	2.007620
35	0	−1.312754	1.312754	−2.007684	2.007684
36	0	−1.312775	1.312775	−2.007716	2.007716
37	0	−1.312786	1.312786	−2.007732	2.007732
38	0	−1.312791	1.312791	−2.007740	2.007740
39	0	−1.312793	1.312793	−2.007744	2.007744
40	0	−1.312795	1.312795	−2.007746	2.007746
41	0	−1.312795	1.312795	−2.007747	2.007747
42	0	−1.312796	1.312796	−2.007748	2.007748
